# Cervico-Ocular and Vestibulo-Ocular Reflexes in Subclinical Neck Pain and Healthy Individuals: A Cross-Sectional Study

**DOI:** 10.3390/brainsci13111603

**Published:** 2023-11-18

**Authors:** Devonte Campbell, Bernadette Ann Murphy, James Burkitt, Nicholas La Delfa, Praveen Sanmugananthan, Ushani Ambalavanar, Paul Yielder

**Affiliations:** Faculty of Health Sciences, Ontario Tech University, Oshawa, ON L1G 0C5, Canada; devonte.campbell@ontariotechu.net (D.C.); james.burkitt@uoit.ca (J.B.); nicholas.ladelfa@ontariotechu.ca (N.L.D.); praveen.sanmugananthan@ontariotechu.net (P.S.); ushani.ambalavanar@ontariotechu.net (U.A.); paul.yielder@ontariotechu.ca (P.Y.)

**Keywords:** cerebellar processing, cervico-ocular reflex (COR), vestibulo-ocular reflex (VOR), subclinical neck pain (SCNP)

## Abstract

Alterations in neck sensory input from recurrent neck pain (known as subclinical neck pain (SCNP)) result in disordered sensorimotor integration (SMI). The cervico-ocular (COR) and vestibulo-ocular (VOR) reflexes involve various neural substrates but are coordinated by the cerebellum and reliant upon proprioceptive feedback. Given that proprioception and cerebellar processing are impaired in SCNP, we sought to determine if COR or VOR gain is also altered. COR and VOR were assessed using an eye-tracking device in 20 SCNP (9 M and 11 F; 21.8 (SD = 2.35) years) and 17 control (7 M and 10 F; 22.40 (SD = 3.66) years) participants. COR gain (10 trials): A motorized chair rotated the trunk at a frequency of 0.04 Hz and an amplitude of 5° while participants gazed at a circular target that disappeared after three seconds. VOR gain (30 trials): Rapid bilateral head movements away from a disappearing circular target while eyes fixated on the last observed target. Independent *t*-tests on COR and VOR gain were performed. SCNP had a significantly larger COR gain (*p* = 0.006) and smaller VOR gain (*p* = 0.487) compared to healthy controls. The COR group differences suggest an association between proprioceptive feedback and SMI, indicating COR may be a sensitive marker of altered cerebellar processing.

## 1. Introduction

When turning the head to view surroundings while crossing the street, or shaking the head contralaterally in response to a question, two oculomotor reflexes interrelate to stabilize vision despite a change in head and trunk position, as well as head movement [1]. The vestibulo-ocular reflex (VOR) and cervico-ocular reflex (COR) stabilize the gaze. They are complementary, with the inactive one suppressed in order to ensure gaze stability is maintained by the active reflex [2] or upregulated in response to disruptions in the other [2]. The VOR stabilizes the gaze during head movements [3], whereas the COR stabilizes the images on the retina by keeping the eyes fixated in the orbits during trunk rotations [4].

Both reflexes rely upon visual, vestibular, and proprioceptive inputs to produce a reflex response [2,5,6,7]. The floccular–nodular lobe of the cerebellum regulates the coordination and modification of the motor response to stabilize the ocular movement [2,5]. When the vestibular system senses movement of the head, it sends afferent signals towards the various vestibular nuclei within the brainstem region, which then project into the cerebellum via mossy fibers [7,8]. Internal cerebellar processing modifies the vestibular input, which then synapses back on the vestibular nuclei, producing a modified signal, which projects to the ocular motor nuclei via the medial longitudinal fasciculus (MLF) [8]. When head-on-trunk or trunk-on-head movement occurs, muscle spindles within the cervical musculature project proprioceptive signals towards the fastigial nucleus within the cerebellum via the spinocerebellar tract. The fastigial nucleus projects this proprioceptive information out of the cerebellum, targeting the four vestibular nuclei which then modify the VOR/COR via the MLF [1]. It is thus likely that altered cerebellar processing would contribute to changes in the stabilization of ocular movement, reflected by functional perturbations within the VOR and COR.

Cerebellar processing can be quantitatively assessed by examining COR and VOR gain. COR gain is the ratio between the peak eye velocity and peak trunk velocity, while VOR gain is the ratio of the peak eye velocity and peak head velocity [9,10].

Previous work suggests that there is COR upregulation in individuals with non-specific neck pain, traumatic and non-traumatic chronic neck pain, and whiplash-associated neck pain [4,9,11,12]. The methodology used in those studies included a bite board to prevent head rotation while the participants were passively moved within an oscillating chair, which may have caused co-activation of the anterior and posterior neck muscles [13]. This approach would also activate other proprioceptive organs within the neck musculature (i.e., muscle spindles and Golgi tendon organs) [14], failing to isolate proprioceptive input from the trunk, and in turn, possibly overestimating the COR response.

Zamysłowska-Szmytke et al. [15] demonstrated COR upregulation in individuals with dizziness, which was accompanied by an asymmetric neck pathophysiology. It is possible that a mismatch between the proprioceptive and vestibular systems from increased neck muscle tension and restricted neck rotation may have contributed to the COR upregulation. This would lead to disruptions within the vestibular system, possibly resulting in a diminished VOR response. However, disruptions within the VOR were not observed in their study [15], nor reported in those with various types of neck pain [4,9,12], despite patients showing an upregulation in COR gain. Such studies may be inherently variable as the VOR response was induced via passive rotation of an oscillating chair at a peak velocity of 5.03°/s while staring at a target. According to the findings of Tabak et al. [16], the VOR is most effective at a higher movement speeds. An active head and neck rotation would permit for higher movement speeds, eliciting a stronger VOR response, as well as replicating a real-world scenario and ensuring the engagement of neck proprioceptors. A longitudinal study conducted by Kelders et al. [17] was the only study to demonstrate COR upregulation with a subsequent decrease in the VOR with age in a population who did not report having pain throughout the study.

VOR and/or COR gain have been assessed in various populations experiencing constant neck pain [4,9,11,12]. Consequently, the presence of pain may have affected neck movement speed, impacting the ability to elicit the VOR. The same could apply for COR, as there is a negative correlation between COR gain and VOR gain [12]. To assess the chronic effects of altered sensory input on COR gain and VOR gain, a population that exhibits altered central processing and also experiences pain-free days is needed.

Individuals with subclinical neck pain (SCNP) have been often used to study altered central processing. SCNP is defined as mild-to-moderate neck pain which has not yet been treated [18]. The recurrent nature of SCNP means that participants can be tested on their pain-free days, when pain does not alter movement patterns, enabling the measurement of central processing changes. Individuals with SCNP exhibit impaired sensorimotor integration due to altered central processing, seen as an altered upper limb joint position sense, altered head and neck movements, and altered glenohumeral and scapular kinematics [19,20,21]. SCNP participants experience altered cortico-cerebellar processing (cerebellar nuclei output towards M1 via the ventrolateral thalamic nucleus), seen as reduced cerebellar disinhibition following motor training in transcranial magnetic stimulation studies [22,23]. Altered inhibitory activity along the olivary–cerebellar pathway is also observed in SCNP participants who acquired either a complex visuomotor task [24] or a proprioceptive-based force-matching task [25], assessed using somatosensory evoked potentials. The effects of these alterations in cortico-cerebellar and central processing on functioning need to be confirmed by assessing the oculomotor reflexes that are known to be coordinated by cerebellum. 

Given that these oculomotor reflexes have not been assessed in an SCNP population, the primary objective of this study was to determine whether there are differences in COR and VOR gain when using protocols that address the limitations of past studies. This study sought to assess the COR and VOR response in those with and without SCNP. It was hypothesized that individuals with SCNP would have an increased COR gain and a diminished VOR gain in comparison to healthy controls.

## 2. Materials and Methods

This was an observational, cross-sectional study design, where COR responses and VOR responses were assessed at one time point in those with and without SCNP. The study was approved by the University’s Research Ethics Board (REB # 14991).

### 2.1. Sample Size

Sample size calculation performed using G*Power 3.1.9.7 statistical software indicated for a large effect size (0.40), an alpha (α) of 0.05, and a power (1–β) of 0.95, a sample size of 15 individuals per group (total sample of 30) would be required.

### 2.2. Participants

Undergraduate students between the ages of 18 and 35 who were attending Ontario Tech University were recruited to participate in this study via verbal and online course announcements. SCNP participants had to have recurring neck pain and/or stiffness for at least three months and not to have received treatment for their neck pain in the past month. All participants were eligible to participate if they met the following conditions: (1) right-hand dominant, (2) no known neurological conditions known to impact cognitive function and/or neural processing (e.g., attention-deficit/hyperactive disorder, multiple sclerosis, stroke, Parkinson’s, head injury with ongoing symptoms), and (3) not taking any medication known to influence neural processing (e.g., antidepressants, neuroleptic or antipsychotic drugs, dopamine partial agonists, cannabis or recreational drugs). Participants were recruited between 7 September 2021 and 30 March 2022.

Eligibility was confirmed using the Von Korff Chronic Pain Grade Scale (CPGS), Edinburgh Handedness Inventory (EHI), and a safety screening checklist. The Von Korff CPGS discriminated SCNP from healthy control participants and has been used in past work to determine the severity, presence, or absence of reoccurring neck pain within the previous six months [26]. SCNP participants had to have a grade between I and III, while healthy participants were to have a grade of 0. The EHI was administered to confirm right-hand dominance for each participant, determined by scores >+40 [27]. A safety checklist was administered to determine if the participant used medication that might alter their balance or alertness (e.g., antidepressants, neuroleptic or antipsychotic drugs, dopamine partial agonists, cannabis or recreational drugs), had a history of neurological disorders (e.g., epilepsy, residual symptoms from traumatic brain injury/concussion in the past five years, etc.), or had impairments in vision (e.g., color blindness) that cannot be corrected by corrective lenses, which could impact the outcome measures.

All 37 participants (20 SCNP and 17 healthy control) who expressed interest were eligible and completed the entirety of the study, which was conducted in a research laboratory at Ontario Tech University. The data collection period was between 24 September 2021 and 6 April 2022. Participants received either a monetary reward of 10 dollars or course credit as compensation for their participation in this study.

### 2.3. Questionnaires: Neck Pain Characteristics

The neck disability index (NDI) was administered to determine neck pain-related disability on the day of the collection [28]. The NDI has been shown to be a reliable and valid measure when administered to groups experiencing neck pain [29,30]. Healthy control participants were to have an NDI score between 0 and 4 (no disability) [30] and SCNP participants were to score less than 15 (acceptable asymptomatic state) [30,31] on the day of collection.

A neck pain visual analog scale (VAS) was also administered on the day of data collection to assess pain levels at the moment of administration [32]. The VAS has been shown to be a valid and reliable tool when administered in groups experiencing various types of neck pain [32,33,34]. Participants were required to have a score of ≤3 cm for the 10 cm VAS, as this is considered the minimum amount of pain, where mobility and/or movement patterns are not impacted via overcompensation [35].

To eliminate the confounding effect of severe pain on movement and neural processing, data collections took place on days when participants were experiencing minimal to no pain.

### 2.4. Instrumentation and Data Acquistion

**Figure 1:**
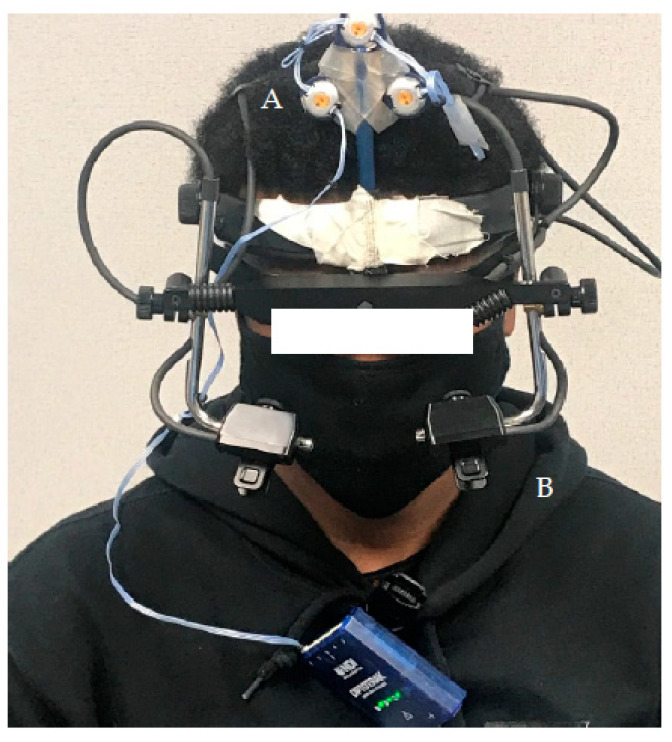
Eye-tracker set-up: (**A**) three infrared markers and (**B**) the eye-tracker used to capture ocular movement.

The Eye-Link-II eye-tracking device (SR-Research, Ottawa, ON, Canada) was utilized to record eye movements during the elicitation of both COR and VOR (see Figure 1). A sampling frequency of 250 Hz [4,9] was used to record monocular eye movement (left eye) during the COR protocol. A sampling frequency of 500 Hz was used to record binocular eye movements during the VOR protocol, as opposed to using a scleral search coil to record monocular (left) eye data [10,36]. Data from both eyes was collected to ensure that the corneal reflection (CR) signal could be tracked on at least one eye when the head was turning to elicit the VOR. The eye-tracking device was fitted with three infrared markers that had a marker power frequency of 1000 Hz. This set-up and the recording of ocular movement were utilized to record the degree of head rotation and angular velocity for each trial for the respective protocols.

A motorized chair that was developed by Sanmugananthan et al. [37] was used in the collection of COR gain in order stimulate the COR response by passively rotating the individual’s trunk. This chair was also fitted with three infrared markers with a marker power frequency of 1000 Hz to record the degree of chair rotation and angular velocity during the protocol. Two Northern Digital Incorporated Optotrak cameras were utilized and sampled at a frequency of 50 Hz during both COR and VOR testing. For the COR protocol, the Optotrak cameras were situated about 275 cm away from the participant to record trunk rotation. For the VOR protocol, the Optotrak cameras were situated about 255 cm away from the participant to record head rotation. The trunk and head rotation data were stored as MATrix LABoratory (MATLAB) files after recording for further analysis.

The ten-point Borg rating of perceived exertion (RPE) scale was used to assess the level of fatigue within the head, neck, eyes, and low back at that instant in time [38] and was administered verbally at various timepoints during the COR protocol.

### 2.5. Experimental Flow

The COR protocol was a modification of existing procedures [4,9,11,17,39,40] while the VOR protocol followed the same protocol as past research [10,36]. The COR protocol was performed before the VOR protocol; details for each respective protocol are provided in subsequent paragraphs. Both COR and VOR were performed in the dark.

#### 2.5.1. COR

Participants were fitted with an Eye-Link-II eye-tracker while they were seated in a custom-built motorized chair that was placed 3 m away from and in front of a fifty-inch monitor with a resolution of 1920 × 1080 pixels. The participant’s head was then secured to the headrest of the chair by tying two cloth straps together to ensure no head movement during passive trunk rotation. This method was chosen because it led to the least amount of neck movement during pilot testing. Following this, the eye position was calibrated using the built-in nine-point calibration system. After calibrating the visual field, participants were instructed to fixate their eyes on the static target projected on the screen; this was done to ensure the system could track the left eye position accurately during each trial.

**Figure 2:**
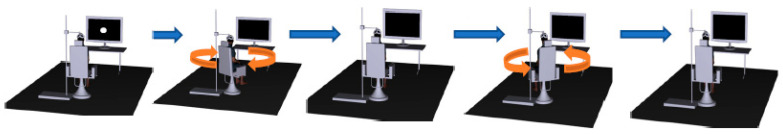
Illustration of the COR Protocol. Orange arrows indicate the direction of chair rotation. Blue arrows indicate progression of protocol, start to finish.

During each trial, participants were required to stare at the circular target that appeared for three seconds and then at its former position after it disappeared (see Figure 2). A disappearing target was chosen because a more prominent COR response is elicited in the absence of visual stimuli [2]. As soon as the circular target appeared, the motorized chair was programmed to rotate the participant’s trunk for five full oscillations at a frequency of 0.04 Hz and an amplitude of 5° over the span of ~120 s. This induced a peak stimulus velocity (i.e., trunk velocity) of approximately 1.5°/s, which occurred when the head and trunk were aligned (i.e., the center position). A total of 10 trials were performed, which took 20 min to complete. This was done to capture the COR gain.

The RPE of the head, neck, eyes, and lower back were recorded at the end of each trial. A mandatory break was provided: (1) if participants reported an RPE > 4 for any of the aforementioned regions following two consecutive trials or (2) after completing the fifth trial. During this break, the participant was unstrapped from the headrest and the eye-tracker was removed from their head.

#### 2.5.2. VOR

**Figure 3:**
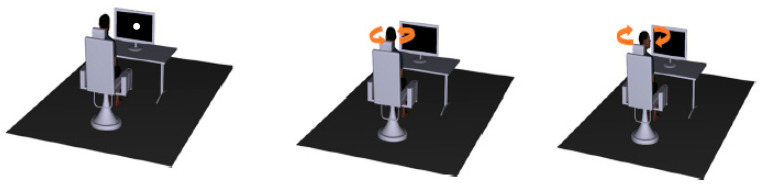
Illustration of the VOR protocol. Orange arrows indicate the direction of head rotation.

Participants were seated 90 cm away from a 26 in monitor (BenQ, Taoyuan, Taiwan) with a resolution of 1920 × 1080 pixels while they were fitted with the Eye-Link-II eye-tracker (Figure 3). Once setup was completed, the eye position was calibrated using a built-in three-point calibration system. Following the completion of the calibration, participants were instructed to fixate their gaze on a fixation target that was projected on the screen in order to accurately fixate the position of the eyes before the trial.

During each trial, participants were required to rotate their head away from the circular target (which appeared after eyes were fixed/recognized by the system and vanished after ± 2° of head rotation) by 15° while keeping their eyes fixated on where they last observed the target. The update rate of the circular target was 11.75 Hz. An end range of 15° was chosen, since pilot testing with an end range of 25° as suggested by past work [36] revealed that participants tended to overshoot the end angle by 5°–10°. Participants were not informed of the speed at which they were to turn to each 15° mark, but they were given auditory feedback regarding the peak angular velocity of their head rotation. Participants heard either: (1) two low-pitched beeps when they were moving too slow (i.e., below 140°/s), (2) two high-pitched beeps if they moved too quickly (i.e., above 170°/s), or (3) a medium-pitched beep when they moved at a speed of 140°/s–170°/s. Once participants reached the 15° mark, they were prompted with a grey screen informing them that they reached the end range. Participants were instructed to stay at that degree of rotation for three seconds, until the grey screen disappeared, before returning to the center position. After returning to the start position, the fixation target reappeared and the next trial began once the circular target was projected on the screen again. Each trial lasted approximately five seconds. A total of 30 trials were performed. If participants turned their head to the right for the first trial, then the subsequent trial was to the left. The starting of rotation to the right or left was counterbalanced between participants. This was performed in order to measure the VOR gain as well as calculate the head peak velocity, variable, and constant error. An active VOR protocol was chosen because this more accurately mimics real world conditions and ensures the involvement of the neck proprioceptors.

Prior to assessing the VOR gain, participants completed 20 practice trials for the purposes of familiarization. During these trials, the fixation target remained in the center of the screen during the first ±2° of head rotation (i.e., target disappearing at +2° of left head rotation and −2° of right head rotation). Upon familiarization, VOR gain was measured using the same parameters.

### 2.6. Data Analysis

All data was analyzed using custom MATLAB R2021a codes/script (The MathWorks Inc., Natick, MA, USA).

#### 2.6.1. COR Analysis

A low-pass, second-order Butterworth filter with a cut-off frequency of 0.5 Hz was applied to the raw chair angular velocity data of each trial, followed by a moving average window of 0.03 s to smooth the signal. Peak chair velocity for each trial was identified and output using the custom MATLAB script.

The custom written MATLAB script also identified and removed blinks, saccades, and fast phases (repetitive, uncontrolled ocular movements) from the raw eye data. A piecewise cubic hermite interpolating polynomial (PCHIP) was then utilized to interpolate and fill in the missing data points within the set. The raw eye angular velocity was filtered utilizing a low-pass, second-order Butterworth filter with a cut-off frequency of 0.25 Hz. A sinusoidal waveform was created for the filtered eye angular velocity data by using an open source MATLAB script provided by Seibold [41]. The amplitude of this sinusoidal waveform was then output for each trial. The formula to calculate the COR gain is as follows:

$$\mathrm{COR}\;\mathrm{Gain}\;=\;\frac{Amplitude\;of\;sinusodial\;waveform}{Peak\;chair\;angular\;velocity}$$

The average of 10 trials was used for statistical analysis.

#### 2.6.2. VOR Analysis

The ipsilateral eye with respect to the head movement was analyzed, i.e., left eye data for left head turns and right eye data for right head turns. Trials from individual participants’ data sets were removed if the following occurred during the trial: (1) the corneal reflection signal was lost during the 120 ms window, (2) the participant did not reach the 15° mark for head rotation, or (3) the participant changed head rotation direction mid trial. On average, 5.5 trials were removed in the healthy control group and 8.5 trials were removed in the SCNP group.

Head displacement and angular velocity signals were filtered using a low-pass, second-order Butterworth filter with a cut-off frequency of 10 Hz. Any missing head angular velocity data points were interpolated utilizing a cubic spline.

Blinks, saccades, and fast phases were all identified and removed. A PCHIP was then utilized to interpolate the slopes of these missing data points. Raw eye displacement and angular velocity signals were filtered with a low-pass, second-order Butterworth filter with a cut-off frequency of 6 Hz. VOR gain was calculated by dividing the average eye velocity by the average head velocity during a 120 ms window, 60 ms before and after peak head velocity.

A 120 ms window was utilized to capture the point at which peak eye velocity occurred. VOR gain was calculated for each trial and averaged.

##### VOR Analysis—Measurement Error

The constant error was calculated as the average error between the target and where the participant landed. The variable error was calculated as variability in their error when comparing their head position to the target position.

### 2.7. Statistical Analysis

All 37 datasets were included in the statistical analysis of COR, NDI scores, and pain VAS scores. Thirty-six datasets were included in the statistical analysis of VOR and the correlation of COR and VOR. Statistical analysis was completed using SPSS version 26 (IBM Corp., Armonk, NY, USA), and statistical significance was set at *p* ≤ 0.05.

The Shapiro–Wilk test was conducted for all dependent variables (COR gain, VOR gain, head peak velocity, constant error, variable error, NDI scores, and pain VAS) to test for normality. Head peak velocity, NDI scores, and pain VAS scores were non-normally distributed while the remaining variables were normally distributed. A log_10_ transformation was applied to normalize the head peak velocity data. A square root transformation was applied to the NDI scores and pain VAS scores to normalize the data. Outliers were removed if a dataset was two standard deviations away from the interquartile range (IQR) of either ± the 75th or 25th percentile. No participants were removed for the COR gain data. A total of 8.3% (three participants (two SCNP and one healthy control)) of the VOR gain data, 2.8% (one healthy control participant) of the variable error, and 2.8% (one SCNP participant) of the NDI and pain VAS score data was removed. Levene’s test of homogeneity was assessed for all variables to determine the equality of variance. COR gain was not equally variant, while all remaining dependent variables had equal variance between the two groups.

Separate independent sample *t*-tests were performed for all dependent variables to determine the differences in means between the two groups. Equal variance was not assumed for COR gain and assumed for the remaining variables. Cohen’s *d* was used to report effect sizes for all dependent variables, where 0.2 is a small effect size, 0.5 is a medium effect size, and 0.8 is a large effect size [42].

In order to correct for multiple hypotheses, the Benjamini–Hochberg test was used [43], which modifies the *p*-value to control for false discoveries. A false discovery rate (proportionate of type I error) was set at a threshold of ≤0.10 [43,44]. The unadjusted *p*-values are reported in the results sections, but it was correction that established the statistical significance of the COR and VOR gain [45].

Pearson’s correlation coefficient was used to assess the relationship between the two reflexes, COR gain and VOR gain. Correlations were run for each group separately.

## 3. Results

Descriptive statistics are reported as mean ± standard deviation.

### 3.1. Demographic and Neck Pain Characteristics

Table 1 provides the demographic and neck pain characteristic of the sample.

### 3.2. COR Gain

**Figure 4:**
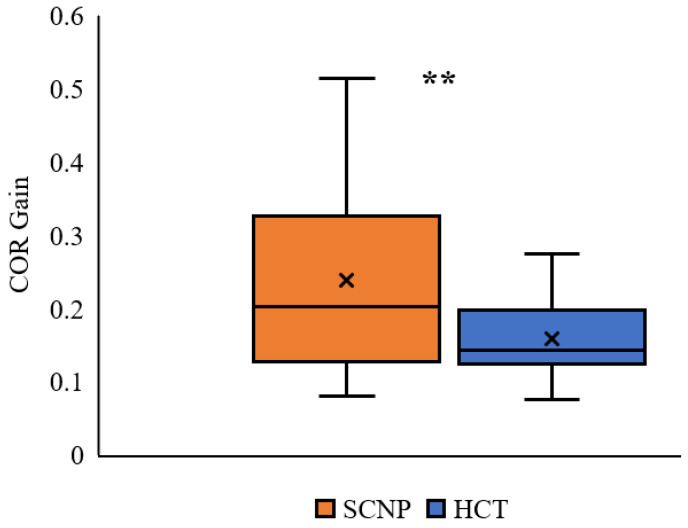
Average COR gain in the SCNP and healthy control (HCT) groups. The whiskers reflect minimum and maximum values. The boxes reflect the quartiles and the × symbol represents the median. ** *p* < 0.01.

COR gain was statistically different between groups, where SCNP participants had a larger COR gain (0.24 ± 0.12) than healthy control participants (0.16 ± 0.05), reflected by a large effect size (T (26.689) = 2.673, *p* = 0.006, *d* = 0.833) (see Figure 4).

### 3.3. VOR Gain

**Figure 5:**
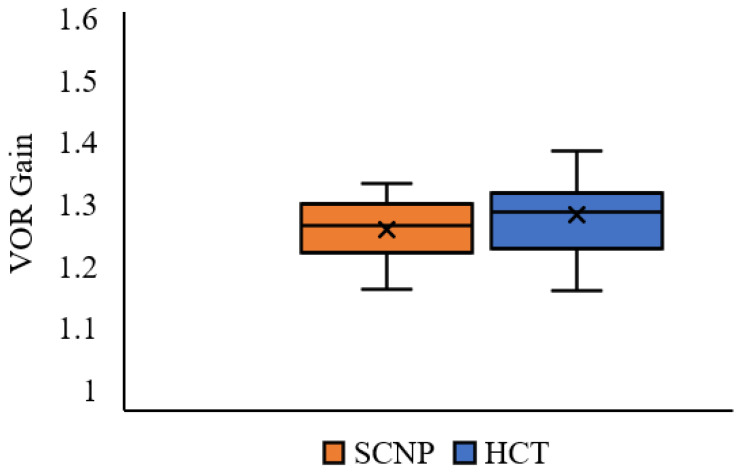
Average VOR gain in the SCNP and healthy control (HCT) groups. The whiskers reflect minimum and maximum values. The boxes reflect the quartiles and the × symbol represents the median.

VOR gain was not statistically different between the groups, where SCNP participants had a slightly lower VOR gain (1.26 ± 0.50) than healthy participants (1.28 ± 0.07), reflected by a near-medium effect size (T (31) = 1.222, *p* = 0.116, *d* = 0.427) (see Figure 5).

#### 3.3.1. Head Peak Velocity

Head peak velocity was not statistically different between the groups, where SCNP participants had a slightly lower head peak velocity (135.88 ± 28.36) than healthy participants (137.96 ± 29.27), reflected by a small effect size (T (34) = 0.154, *p* = 0.439, *d* = 0.051).

#### 3.3.2. Measurement Error

##### Constant Error

Constant error was not statistically different between the groups, where SCNP participants had a slightly lower directional bias (13.93 ± 4.39) than healthy participants (14.79 ± 4.47), reflected by a small effect size (T (34) = 0.579, *p* = 0.283, *d* = 0.193).

##### Variable Error

Variable error was not statistically different between the groups, where SCNP participants had a slightly greater variability in error (3.43 ± 0.83) than healthy participants (3.40 ± 0.84), reflected by a small effect size (T (33) = 0.093, *p* = 0.463, *d* = 0.032).

### 3.4. Correlation of COR Gain and VOR Gain

**Figure 6:**
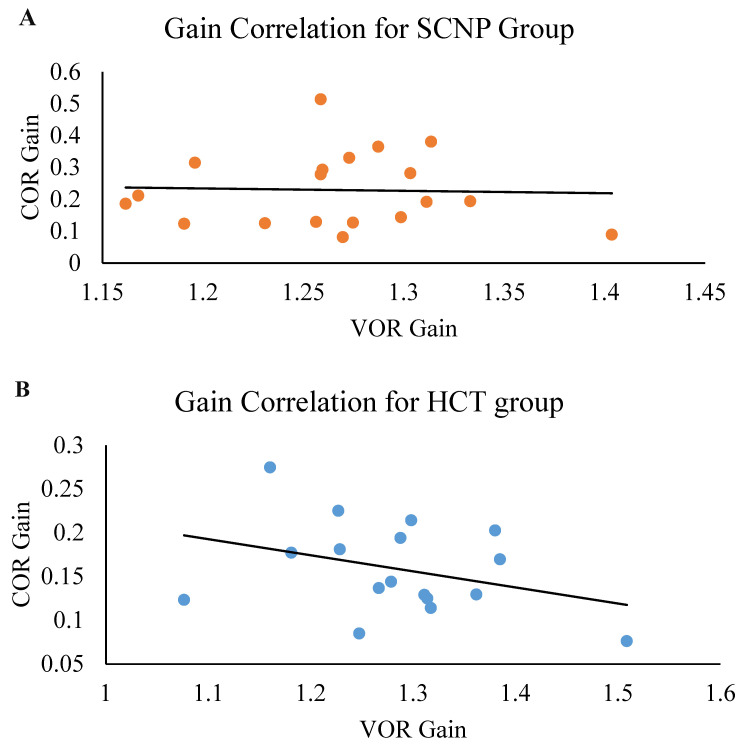
Correlation plot comparing the VOR gain (*x*-axis) to the COR gain (*y*-axis) in the (**A**) SCNP and (**B**) healthy control (HCT) groups.

There was no significant correlation between COR gain and VOR gain in the SCNP group (r = −0.038, *p* = 0.438) nor in the healthy control group (r = −0.350, *p* = 0.088) (see Figure 6).

## 4. Discussion

This is the first study to use oculomotor reflexes (VOR and COR) as a tool to examine cerebellar mechanisms in a population with SCNP. This study demonstrated that there are differences in the circuitry pertaining to proprioceptive feedback on image stabilization during trunk-on-head rotations, seen as greater COR gain in the SCNP group compared to healthy controls. The lack of group differences in VOR gain could be the VOR’s reliance on multiple sensory inputs. The findings suggest that COR might be a more sensitive measure of altered cerebellar processing in a population with recurrent neck pain.

### 4.1. COR Gain

The increased COR gain in the SCNP population corresponds with the literature that has examined COR gain in those with non-specific neck pain [4], traumatic and non-traumatic chronic neck pain [9], and whiplash-associated neck pain [11,12] using a bite board. The current work is also in line with Zamysłowska-Szmytke et al. [15], who found an upregulation of the COR when the head was immobilized manually in individuals with an asymmetric neck pathophysiology in conjunction with dizziness. The mean COR gain of the healthy control group is in line with past work that asserts that COR gain in a healthy population may be as low as 0.1 or completely absent [4,46]. The differences in COR gain in a population with subclinical pain suggests that the protocol that was implemented to mitigate challenges with past studies (such as unintended proprioceptive input from the neck when using a bite board or manual head immobilization) [4,9,11,12,15] was sensitive enough to demonstrate differences in COR gain in those with SCNP relative to healthy controls. The findings in this study expand on past work that suggests COR upregulation may be the result of altered muscle spindle activity from neck pain.

The group differences in COR gain may be reflective of alterations in proprioceptive input, as this oculomotor reflex is heavily dependent on input from the proprioceptive system [2], particularly in muscle spindles within the cervical musculature [4]. The muscle spindles located within the deep neck musculature relay proprioceptive information essential to eye and head coordination [47,48,49,50], and the muscle spindle feedback from the cervical region (high density of muscle spindles located here) plays a key role in the integration of the head and neck position [47]. This suggests that alterations in muscle spindle activity in response to altered afferent input from the neck may impact the coordination of eye movements during trunk rotation. Röijezon et al. [51] suggested that proprioceptors located in the neck detect changes in head and neck velocity during trunk-on-head rotations, which are transmitted along the spinocerebellar tract. The spinocerebellar tract relays unconscious proprioceptive information from muscle spindles, Golgi tendon organs, and joint capsules to the cerebellum via structures in the spinal cord and brainstem [51]. The proprioceptive input from the trunk is transmitted along the posterior spinocerebellar tract via the inferior cerebellar peduncle, while some of this proprioceptive information (acquired during head rotation) is transmitted to the flocculus of the cerebellum via mossy fibers from the central cervical nucleus [2]. This information is processed in the cerebellum, and the COR response is modified by floccular Purkinje cells via inhibitory signals to the vestibular nuclei to meet the demands of the trunk-on-head movement [2,52]. Based on the neural mechanism of COR, it is possible that an alteration in proprioceptive input due to altered central processing and/or neck sensory input may lead to an upregulation in COR.

It is also likely that the ability to formulate and maintain the internal representation of the body (i.e., body schema) would also be impacted by altered proprioceptive input [53]. An altered body schema would also explain past work on individuals with SCNP, as this population exhibits impairments in proprioceptive awareness of the head and neck [54], elbow [19], and head-on-trunk [21], as well as an inability to adapt to perturbations [53,55]. Researchers have theorized that an alteration in muscle spindle discharge may alter proprioceptive input [21]. The upregulation in COR in the SCNP group could be the result of inaccurate somatosensory information in response to chronic changes in muscle spindle input [56], suggesting that altered proprioceptive input may be the culprit for disordered cerebellar processing in this population. Thus, altered proprioceptive input and feedback from a region with a high density of muscle spindles is likely to impact eye and head coordination [47,48,49,50].

### 4.2. COR and VOR Gain

There was no correlation between VOR gain and COR gain in the SCNP group and a moderate negative correlation in the healthy control group. Research has indicated that an inverse relationship exists between these two oculomotor reflexes, where a suppression in VOR would yield an upregulation in COR (especially when there are disruptions in the VOR), and vice versa [12,15,57]. This trend is apparent in the control group. The uncoupling of the relationship between VOR and COR in the SCNP group may be related to sensory mismatches between proprioceptive input from the neck and vestibular input, as seen in past work [4]. The COR and VOR share many of the same neural circuits for their respective roles in vision stabilization [2]. It is plausible that chronic alterations in proprioceptive input from recurrent neck pain may not impact the VOR greatly since it also heavily relies on visual and vestibular input, whereas the COR relies more on proprioceptive input [2]. Past work has yet to find a downregulation in the VOR response despite showing an upregulation in COR gain in various neck pain populations, such as those with non-specific neck pain, traumatic and non-traumatic chronic neck pain, and whiplash-associated neck pain [4,9,11,12]. As the past literature has suggested, traumatic forms of neck pain that have impacted the vestibular system and their associated neural substrates, seen as dizziness and visual disturbances [58], are likely to result in a downregulation in VOR gain. It is possible that SCNP is not severe enough to impact the neural networks pertaining to VOR, reflected by the lack of an inverse relationship in this group.

### 4.3. VOR Gain

In comparison to the healthy control group, the SCNP group had a smaller VOR gain. This reduced VOR gain contradicts the literature that has examined VOR gain in populations with non-specific neck pain [4], non-traumatic chronic neck pain [9], and neck pain from whiplash injury [40]. Those past studies demonstrated similar VOR gains between groups, which were lower than one. However, those studies used a passive VOR protocol which was performed at a slower speed (5.03°/s) instead of a higher speed (e.g., 140–170°/s). Despite the use of an active protocol that was performed at a higher speed, the lack of group differences in VOR gain in this study corresponds with past work [4,9,40]. The VOR gain response greater than one observed in both groups coincides with work conducted by Fadaee et al. [10]. Fadaee et al. [10] demonstrated that a greater VOR gain is observed at baseline when a lower frequency such as 15 Hz is utilized to update the presentation of the visual target, whereas this study had an update rate of 11.75 Hz. This similar increase in VOR response alongside fairly similar head peak velocities suggests that the subtle group differences observed are attributable to changes within the neural processing of the VOR. This finding expands on past work that suggests there may be a compensatory effect in the VOR, resulting in a lack of group differences in a population with neck pain, irrespective of type.

The lack of group differences in VOR gain may suggest that subclinical neck pain has little impact upon the vestibular input towards the cerebellum. As previously mentioned, the COR and VOR have similar neural mechanisms pertaining to proprioceptive input despite having different roles with respect to image stabilization. The VOR relies more on vestibular input, where sensory information is transmitted from the vestibulocochlear nerve to the four vestibular nuclei as well as the cerebellum via the inferior cerebellum peduncle [7,8]. The cerebellum then makes adjustments to the motor output of the muscles by sending descending signals via the medial vestibulospinal tract and lateral vestibulospinal tract to coordinate the orientation of the neck musculature and the limbs, respectively [8]. Sensory feedback from the medial and inferior vestibular nuclei to the cerebellum is also used to modify activity of the vestibular nuclei and transmitted to the three oculomotor nuclei via the medial longitudinal fasciculus [7,8]. These pathways work in conjunction with the cerebellum to modify the ocular movement/velocity of the eyes and head during the VOR response [7,8]. It is likely that SCNP may not have a significant impact on the cerebellum’s ability to process vestibular information since the VOR has numerous inputs which are able to compensate for possible alterations in neck proprioceptive feedback. This is reflected by the lack of group differences in both the constant and variable error, which are measures used to assess proprioceptive deficits. This is in line with the current literature, which has mainly found alterations within the VOR following various changes within the vestibular system such as bilateral vestibular labyrinth lesions and a diminished vestibular system as a secondary consequence of aging [17,57], rather than following alterations in the neck proprioceptors [4,9,15].

### 4.4. Limitations

The findings of this study are currently only applicable to a university-aged population, as these oculomotor reflexes change with age. Despite trying to minimize the impact of neck fatigue and discomfort while wearing the eye tracker and restricting head movement in the COR protocol, it is possible such discomfort may have aggravated SCNP participants’ sensitivity, inducing acute pain and predisposing associated altered proprioceptive input from the neck musculature. Neck fatigue could have carried over to the VOR since the COR was measured first, and the same is true with respect to attention levels. The level of fatigue was controlled by administration of the Borg’s RPE scale to monitor the participants’ perceived level of fatigue within their neck and to ensure fatigue-induced pain was not a limiting factor. The current study did not screen healthy control participants for vestibular disturbances (e.g., motion sickness), but the participants who took part in this study did not demonstrate any underlying vestibular alterations based on VOR gain. Given that this is an observational study, it is possible that unmeasured unconfounding variables (e.g., past sporting or concussion history beyond five years, etc.) could impact the interpretation of the findings. Additionally, the study was powered for a large effect size. Therefore, it is possible that a large sample size might have shown small or medium effects.

### 4.5. Future Studies

Subsequent studies could consider including individuals with and without vestibular issues in conjunction with SCNP to determine whether a vestibular disturbance alongside an alteration in central processing from SCNP leads to a VOR downregulation alongside a COR upregulation. A measure to assess correlations between the COR and neck proprioception would be of interest to confirm that the body schema is impaired and may be another neurophysiological mechanism and to confirm the trends in this study. Future research could consider using glasses that permit the measurement of the VOR and the COR, as the current eye-tracking system was quite heavy and may have introduced fatigue despite trying to minimize it during the protocol.

## 5. Conclusions

This study demonstrates that altered proprioceptive input from SCNP upregulates the COR, likely as a result of altered cerebellar processing from proprioceptive feedback. This suggests that there may be an association between altered proprioceptive feedback and disordered sensorimotor integration in this population, as there is minimal impact on the processing of vestibular input.

## Figures and Tables

**Table 1:** Baseline characteristics of the sample.

		**SCNP Group**	**Healthy Control Group**	
Biological Sex (F:M)		11:9	10:7	
Age (years)		21.80 ± 2.35	22.40 ± 3.66	
Von Korff CPGS				
	Grade 0	0	17	
	Grade I	13	0	
	Grade II	6	0	
	Grade III	1	0	
	Grade IV	0	0	
		SCNP Group	Healthy Control Group	*p*-value
* NDI Score (/50)		7.95 ± 5.53	0.77 ± 1.09	<0.001
* Pain VAS (/10 cm)		1.47 ± 1.05	0.14 ± 0.18	<0.001

* Note: Measured on the day of testing.

## Data Availability

The datasets generated during and/or analyzed during the current study are available from the corresponding author on reasonable request.
